# Multiple sex chromosomes in teleost fishes from a cytogenetic perspective: state of the art and future challenges

**DOI:** 10.1098/rstb.2020.0098

**Published:** 2021-09-13

**Authors:** Alexandr Sember, Petr Nguyen, Manolo F. Perez, Marie Altmanová, Petr Ráb, Marcelo de Bello Cioffi

**Affiliations:** ^1^ Laboratory of Fish Genetics, Institute of Animal Physiology and Genetics, Czech Academy of Sciences, Rumburská 89, 277 21 Liběchov, Czech Republic; ^2^ Faculty of Science, University of South Bohemia, Branišovská 1760, 370 05 České Budějovice, Czech Republic; ^3^ Departamento de Genética e Evolução, Universidade Federal de São Carlos, Rod. Washington Luiz km 235 cep, 13565-905, São Carlos, Brazil; ^4^ Department of Ecology, Faculty of Science, Charles University, Viničná 7, 128 44 Prague, Czech Republic

**Keywords:** chromosome rearrangements, fish, repetitive DNA accumulation, sex chromosome differentiation, sex chromosome turnover

## Abstract

Despite decades of cytogenetic and genomic research of dynamic sex chromosome evolution in teleost fishes, multiple sex chromosomes have been largely neglected. In this review, we compiled available data on teleost multiple sex chromosomes, identified major trends in their evolution and suggest further trajectories in their investigation. In a compiled dataset of 440 verified records of fish sex chromosomes, we counted 75 multiple sex chromosome systems with 60 estimated independent origins. We showed that male-heterogametic systems created by Y-autosome fusion predominate and that multiple sex chromosomes are over-represented in the order Perciformes. We documented a striking difference in patterns of differentiation of sex chromosomes between male and female heterogamety and hypothesize that faster W sex chromosome differentiation may constrain sex chromosome turnover in female-heterogametic systems. We also found no significant association between the mechanism of multiple sex chromosome formation and percentage of uni-armed chromosomes in teleost karyotypes. Last but not least, we hypothesized that interaction between fish populations, which differ in their sex chromosomes, can drive the evolution of multiple sex chromosomes in fishes. This underlines the importance of broader inter-population sampling in studies of fish sex chromosomes.

This article is part of the theme issue ‘Challenging the paradigm in sex chromosome evolution: empirical and theoretical insights with a focus on vertebrates (Part II)’.

## 1. Introduction

The classical model of sex chromosome evolution posits that they evolved from a pair of autosomes that acquired a sex-determining factor. Sexually antagonistic selection favours the restriction of recombination between the sex-determining and sexually antagonistic loci, which results in the degeneration of sex-specific chromosome Y or W in male- and female-heterogametic systems, respectively. This theory is championed especially by studies of eutherian mammals and *Drosophila* spp. In the latter, the neo-sex chromosomes formed by sex chromosome–autosome fusions of various ages provided an insight into the distinct stages of the differentiation process, which is otherwise difficult to study in evolutionarily old systems [1]. However, the classical paradigm has recently been challenged by examples in other species, most directly in cold-blooded vertebrates [2–5].

Teleost fishes encompass more than half of the extant vertebrate biodiversity [6,7], making this group extremely attractive for studying a variety of evolutionary subjects, including genome and karyotype evolution. They also represent one of the most diverse animal groups in terms of sex determination and differentiation [8–13]. Fish sex chromosomes typically represent early phases of differentiation with no pronounced changes in their morphology, size and genetic content [8,9,14], although there are exceptions particularly in Neotropical fishes (e.g. [15–20]). This could be owing to the high plasticity of fish sex chromosomes and their frequent turnovers, which repeatedly reset the process of sex chromosome differentiation. This further facilitates the self-enforcing loop between low degeneration and successive turnovers [9,14,21–23].

Nine types of cytogenetically distinct sex chromosome systems with either male or female heterogamety at various stages of their differentiation have been reported in only approximately 5% of the cytogenetically analysed teleosts (based on Arai [24]). However, recent genomic approaches have allowed the identification of small sex-determining regions in many fish species [10,14] using either segregation of single-nucleotide polymorphisms (SNPs) in the F_1_ progeny or the association of SNPs with heterozygous sex [25]. Genomic studies point to the presence of homomorphic, i.e. cytologically indistinguishable, sex chromosomes in teleosts and suggest that their incidence is grossly underestimated [14,25–27].

Besides standard constitutions (♀XX/♂XY; ♂ZZ/♀ZW), fish sex chromosomes include derived systems in which the Y or W chromosome has been lost, i.e. ♀XX/♂X0, ♂ZZ/♀Z0, as well as multiple sex chromosome systems ♀X_1_X_1_X_2_X_2_/♂X_1_X_2_Y, ♀XX/♂XY_1_Y_2_, ♀X_1_X_1_X_2_X_2_/♂X_1_Y_1_X_2_Y_2_, ♂ZZ/♀ZW_1_W_2_, ♂Z_1_Z_1_Z_2_Z_2_/♀Z_1_W_1_Z_2_W_2_ (e.g. [28–31]). Multiple sex chromosomes may correspond to: (i) systems with polygenic sex determination, in which alleles from multiple unlinked loci determine sex (based on a cumulative effect) or different sex chromosomes compete in an ephemeral transitional stage during sex chromosome turnover, where one system is epistatically dominant to the other [10,32–34]; (ii) neo-sex chromosome systems which result from rearrangements between ancestral sex chromosomes and autosomes [29,35–38]; and (iii) systems resulting from fissions of the ancestral sex chromosome pair, without the involvement of new autosomal material [39–41].

In the present review, we summarize the current knowledge of multiple sex chromosomes in teleosts and provide new insights into general patterns of their emergence and evolution.

## 2. Multiple sex chromosomes and their importance for research of fish sex chromosomes

In lineages with old and highly degenerate sex chromosomes e.g. mammals and some birds [4,42,43], little can be learned about the factors and mechanisms behind suppressed recombination and sequence divergence between the sex chromosomes, therefore the investigation of younger autosomal additions to sex chromosomes is vital for this type of study [44,45]. In fishes, however, both standard and multiple sex chromosomes often display a low degree of differentiation [8,9]. The analysis of the subtle differences between these sex chromosomes may provide key insights into the evolutionary processes operating at early phases of differentiation [9]. Furthermore, teleosts and ray-finned fishes, in general, encompass various forms of genetically or environmentally driven sex determination (and the continuum between the two), which can substantially differ between closely related taxa [8–11].

Multiple sex chromosomes can be easily detected by light microscopy as they usually result in different chromosome numbers between sexes [35] and one sex chromosome often notably differs in size and/or morphology. This facilitates more thorough sex chromosome investigations in particular teleost groups, as is the case with sticklebacks (Gasterosteidae)*,* which represent the most comprehensively studied teleost taxon concerning the evolution and differentiation of multiple sex chromosomes. This group encompasses three different sex chromosome systems: XY, ZW and X_1_X_2_Y. The latter formed independently in two species *via* sex chromosome–autosome fusions [46,47]. In *Gasterosteus nipponicus*, the fusion brought under sex linkage the genes important for sexual dimorphism and mating behaviour, contributing to its reproductive isolation with its sister species *Gasterosteus aculeatus* [46]. Numerous studies have extended our understanding of sex chromosome differentiation in this model system (for details, see §4a) and showed that multiple sex chromosomes are an extremely interesting and important subject in the systematic investigation of fish sex chromosomes. Yet this has been largely untapped with the exception of two recent reviews compiling information on multiple sex chromosomes in fishes [29,41].

## 3. Multiple sex chromosome constitutions and their distribution in teleosts

**Figure 1.**
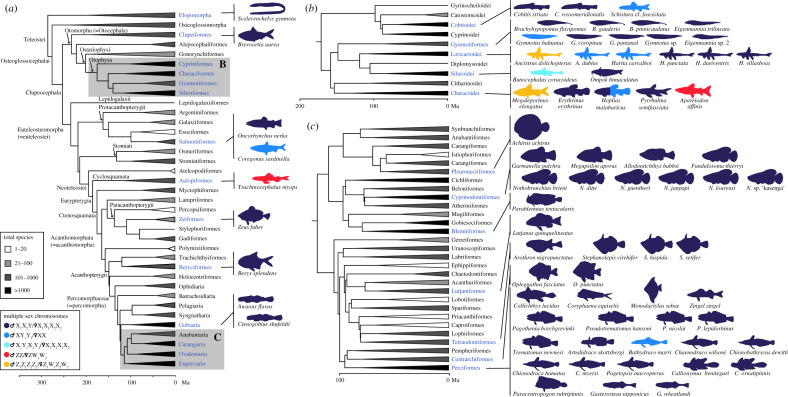
Distribution of multiple sex chromosomes across the teleost phylogeny. (The simplified phylogeny of Teleostei is based on and modified from Betancur-R *et al*. [48]. The types of multiple sex chromosomes are distinguished based on the colour of fish silhouettes.)

Previously, 47 cases of multiple sex chromosomes in teleosts were recorded, including populations of the same species with different sex chromosome systems [29,41]. The present updated dataset encompasses 75 cases (figure 1; electronic supplementary material, table S1).

**Figure 2.**
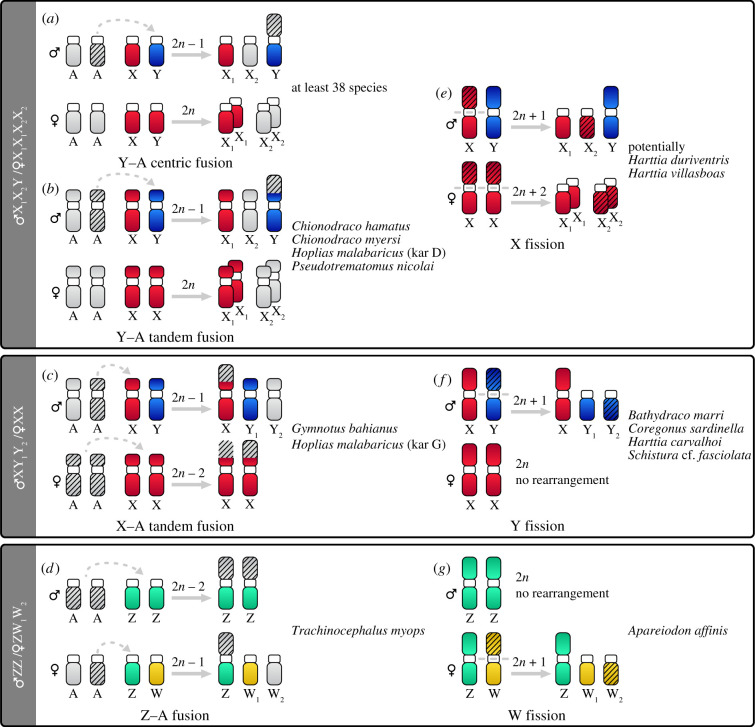
Mechanisms underlying multiple sex chromosome formation in fishes. Seven types of chromosome rearrangements are known to be involved in emergence of the following multiple sex chromosome systems in teleosts: ♀X_1_X_1_X_2_X_2_/♂X_1_X_2_Y, ♀XX/♂XY_1_Y_2_ and ♂ZZ/♀ZW_1_W_2_. The mechanisms behind the origin of two remaining systems (♀X_1_X_1_X_2_X_2_/♂X_1_Y_1_X_2_Y_2_ and ♂Z_1_Z_1_Z_2_Z_2_/♀Z_1_W_1_Z_2_W_2_) need yet to be thoroughly explained. (*a*) Centric fusion: two uni-armed chromosomes are fused at their centromeric regions to form a larger bi-armed element. (*b,c*) Tandem fusion: a centromere of one chromosome is fused to a telomere of another chromosome, giving rise to a larger element (herein a bi-armed one). (*e–g*) Centric fission: characterized by a single break in the centromeric region of a bi-armed element, giving rise to two smaller uni-armed chromosomes. The mechanisms are herein presented in the context of an autosome pair (marked as ‘A’) from which one homologue is fused to a member of standard sex chromosome pair (XY or ZW). The elements undergoing fusion are marked by an arrow (*a–c*). In the case of centric fission (*e–g*), the region of breakage on the sex chromosome is marked by a dashed line. Except for the most prevalent system (Y–A centric fusion), examples of teleost taxa are given to each type of mechanism (a detailed list of all teleost taxa possessing multiple sex chromosomes is given in the electronic supplementary material, table S1). The resulting sex-specific change in diploid chromosome number (2*n*) is indicated. In the case of Z–A fusion (*d*) more specific details about the mechanism are lacking.

The most prevalent multiple sex chromosome system is ♀X_1_X_1_X_2_X_2_/♂X_1_X_2_Y (*n* = 63; electronic supplementary material, table S1). Among teleosts, it was first described in the monotypic Mexican cyprinodontid pupfish *Megupsilon aporus* [49], a species now considered extinct in the wild [50]. The common feature of this system is the presence of a conspicuously large bi-armed (i.e. metacentric or submetacentric) chromosome exclusive to males. This male-limited sex chromosome is usually formed by a centric or tandem fusion of the ancestral Y with an autosome (Y–A fusion; figure 2*a,b*), giving rise to a so-called neo-Y chromosome. However, the X_1_X_2_Y system could have also originated from the fission of an ancestral X (figure 2*e*), although this scenario has been among teleosts only recently proposed in two armoured catfishes of the genus *Harttia* [51]. Another option would be a reciprocal translocation between the ♂X0/♀XX system and an autosome pair. However, the resulting neo-Y chromosome would not be conspicuous in size. A comparison of the diploid chromosome number (2*n*) and karyotype structure between closely related species which do not possess multiple sex chromosomes is critical for distinguishing between the above mechanisms (figure 2; [29]), and this also applies to the assessment of the mechanisms discussed below.

Much less frequent (*n* = 7) is the second male-heterogametic system ♀XX/♂XY_1_Y_2_. It can be formed either by a centric or a tandem fusion between an ancestral X chromosome and an autosomal homologue (X-A fusion; figure 2*c*), as observed e.g. in one karyotype form of the erythrinid wolf fish *Hoplias malabaricus* [52], or by the fission of the original Y chromosome (figure 2*f*). The latter mechanism seems to be more common (described in four species so far; [53–56]).

A very unusual type of multiple sex chromosome system was documented in the asprenidid banjo catfish *Bunocephalus coracoideus*, where a different karyotype composition between males and females with the same 2*n* = 42 suggested the ♀X_1_X_1_X_2_X_2_/♂X_1_Y_1_X_2_Y_2_ system [30]. Similar constitutions observed in plants and other vertebrates [37,57,58] were attributed to reciprocal translocations. In *B. coracoideus*, however, crossing between individuals of chromosomal races was proposed. Unfortunately, the analysis of meiotic pairing, which would test for the expected presence of the sex chromosome quadrivalent, has yet to be carried out. A high rate of chromosome rearrangements was observed on the inter-population level in this species and the ♀X_1_X_1_X_2_X_2_/♂X_1_Y_1_X_2_Y_2_ system has not been found in any other populations of this putative species complex [59].

Comparably rare is the female-heterogametic system ♂Z_1_Z_1_Z_2_Z_2_/♀Z_1_W_1_Z_2_W_2_ found only in the loricariid catfish *Ancistrus dolichopterus* [28,60] and the anostomid characin *Megaleporinus elongatus* [61,62]. This multiple sex chromosome system has almost no parallel in other organisms with female heterogamety except for butterflies, in which complex derivatives also exist [63].

The second and only other female-heterogametic multiple sex chromosome system reported in fishes is ♂ZZ/♀ZW_1_W_2_, found in the marine lizardfish *Trachinocephalus myops* [64] and the parodontid characin *Apareiodon affinis* [39,65]. While in *T. myops*, the proposed mechanism of origin is a Z–A fusion [64] (figure 2*d*), in *A. affinis* it is thought that it emerged from a centric fission of an ancestral W chromosome (figure 2*g*) followed by additional rearrangements [39,65]. In *T. myops*, the sex chromosomes are highly differentiated as neo-Z is disproportionally large in comparison to its small-to-tiny W_1_ and W_2_ counterparts [64], or, alternatively, the mechanism of their origin was more complex.

Fish sex chromosomes evolved independently in many taxa, sometimes even within the same genus or species [21,66]. Our compiled dataset mapped together with numbers of taxonomically recognized species (electronic supplementary material, table S2, [7]) on the teleost phylogeny (figure 1; [48]) indicates that multiple sex chromosomes are scattered across the entire teleost clade. From a broader perspective, no multiple sex chromosomes have so far been reported for any of non-teleost ray-finned fishes (Actinopterygii) [13,24], whereas X_1_X_2_Y sex chromosomes have been found in cartilaginous fishes (Chondrichthyes) [67,68]. Although sampling bias cannot be ruled out, there are no reports of multiple sex chromosomes in three speciose teleost orders (Stomiiformes, Gadiformes or Ophidiiformes), while these are common in other lineages (electronic supplementary material, table S2). Considering the proportion of species with multiple sex chromosome systems relative to the overall number of species with sex chromosomes in orders where these data are available for more than 10 species (electronic supplementary material, Appendix S1 and table S3), we observed a significantly higher percentage of multiple sex chromosome systems in the order Perciformes (60%; Fisher's exact test *p* = 0.012) compared to the mean (17.9%) across all orders. Within Perciformes, 66.7% of the cases belong to the Antarctic sculpin clade Notothenioidei (namely families Artedidraconidae, Bathydraconidae, Channichthyidae and Nototheniidae; [69,70]). The other orders do not differ significantly from the average. At the genus level, the highest number of multiple sex chromosomes (six) has so far been reported in African annual killifish *Nothobranchius* (figure 1; electronic supplementary material, table S1; [71,72]). Some not well supported or even inconsistent reports, such as in the goby *Awaous flavus* and the doryfish *Zeus faber* (electronic supplementary material, table S1), would benefit from a re-examination, as well as several other speculative cases of putative sex-linked chromosome polymorphisms reported, e.g. in the salmonids *Coregonus albula* [73] and *Salvelinus alpinus* [74].

An important caveat to the present dataset is that more than half of the records are based only on conventional cytogenetic methods, i.e. uniform staining of chromosomes (typically by Giemsa solution), supplemented in some cases by differential staining of constitutive heterochromatin (C-banding) producing characteristic bands (electronic supplementary material, table S1). These methods may determine chromosome counts and morphology; C-banding may additionally reveal differences in heterochromatin distribution between sex chromosomes. However, the accurate identification of all elements of the multiple sex chromosome constitution is often challenging. For example, it is difficult to identify individual uni-armed chromosomes such as subtelocentrics and acrocentrics in karyotypes consisting predominantly of uni-armed elements gradually decreasing in size, hence, X_1_ and X_2_ chromosomes in the X_1_X_2_Y system are often chosen arbitrarily. Also, without the analysis of meiotic pairing, we cannot reliably differentiate between XX/X0 and X_1_X_2_Y systems, which form a univalent and a trivalent, respectively, in the first meiotic division, as male and female 2*n* differ in the same way in both constitutions. If data on meiotic pairing are not available, 2*n* and karyotype structure should be compared with several closely related species in order to determine whether chromosome rearrangements such as fusions or fissions have occurred (e.g. [53,75]; figure 2). In addition, the analysis of multiple sex chromosome pairing in meiosis could reveal regions of asynapsis (i.e. mispairing between homologues) as a sign of sex chromosome differentiation [76], which may be further characterized by immunostaining specific proteins of the synaptonemal complex [77]. Numerous studies in teleosts also do not distinguish ancestral sex chromosome elements from the new additions. Chromosome landmarks such as a characteristic block of constitutive heterochromatin [54,78,79] or accumulations of specific repetitive elements traced by fluorescence *in situ* hybridization (FISH) (e.g. [52,55,69,80–82]) are helpful, especially if they highlight a putative region of sex chromosome differentiation. However, as fish multiple sex chromosomes do not display pronounced degeneration, these markers are of limited use and hence a suite of advanced molecular cytogenetic techniques must be used to improve chromosome identification (see §§4a and 4c for details).

Moreover, it has been repeatedly shown that populations of many teleost species and species complexes are polymorphic for sex chromosome systems [31,59,65,70,79,83–89]. These observations point to a very recent, ongoing and recurrent formation of multiple sex chromosomes and stress the importance of proper sampling and sample size. In fishes, initial reports of multiple sex chromosomes have already been refuted using larger sampling [90]. As highlighted in the electronic supplementary material, table S1, around half of the studies had been conducted on a single population, some with only a rather limited sampling.

## 4. Evolutionary pathways of teleost multiple sex chromosomes: current state of knowledge and further research directions

The number of reports of multiple sex chromosomes in teleosts is ever-growing. Despite our increasing knowledge of mechanisms of their origin in particular lineages, it is yet to be elucidated: (i) what forces drive the evolution of multiple sex chromosomes, (ii) whether it is associated with changes in the sex-determining pathway, and (iii) whether they contribute to species diversification.

### (a) Genetic content of fish multiple sex chromosomes

Despite some exceptions (see below), it has been repeatedly documented that fish multiple sex chromosomes do not accumulate heterochromatin [17,91,92]. However, how this relates to levels of their differentiation is currently unclear (cf. [93]). Sex chromosome differentiation reflects recombination, yet the empirical data about the recombination landscape of fish multiple sex chromosomes are so far limited solely to sticklebacks [46,47,91,94–96].

Although the absence of substantial blocks of constitutive heterochromatin simplifies sequencing and assembly of unpaired sex chromosomes, male-limited neo-Y chromosomes have only been sequenced in a few species so far. In the spinyhead croaker *Collichthys lucidus*, the recent report identified *dmrt1* as a candidate master sex-determining (MSD) gene [97], while in the barred knifejaw *Oplegnathus fasciatus* [98], a comparison between the male and female assemblies revealed approximately 99% identity between the neo-Y and the X_1_ and X_2_ sex chromosomes, several rearrangements in the interstitial regions of the neo-Y, and a set of male-specific genes. The extent of neo-Y differentiation has been thoroughly studied in the sticklebacks *G. nipponicus* [95,96] and *Gasterosteus wheatlandi* [94]. In the X_1_X_2_Y system of *G. nipponicus* formed within the last 2 Myr (million years) by a Y–A fusion, recombination between the new sex-linked regions ceased gradually, starting from the fusion point and spreading across a large region of the neo-Y [91]. No clear signs of degeneration of the neo-Y were observed, although its genes started accumulating deleterious mutations [96]. In *G. wheatlandi*, the X_1_X_2_Y system of comparable age evolved independently *via* fusion of the ancestral Y chromosome with another autosome. The *G. wheatlandi* neo-Y underwent also little degeneration, though its non-recombining region is much larger. Interestingly, the shared ancestral Y chromosome experienced more extensive differentiation in *G. wheatlandi* than in *G. aculeatus* and *G. nipponicus* [94].

As for a putative MSD gene, *G. nipponicus* and *G. wheatlandi* share an ancestral Y sex chromosome with the three-spined stickleback, *G. aculeatus*, in which the *amhy* gene was identified as a potential MSD candidate [94,99]. Similarly, there are strong indications that the neo-Y of sockeye salmon *Oncorhynchus nerka* contains *sdY* as a salmonid-specific MSD gene [100–102]. However, the candidates for MSD function in other teleost fishes with multiple sex chromosomes currently remain unknown.

Despite recent progress in fish genomics, our knowledge of the genetic content of fish multiple sex chromosomes still largely stems only from cytogenetic analyses detecting, to various degrees, differences in molecular composition. Several repetitive DNA sequences were mapped on fish multiple sex chromosomes, which helped to determine their evolutionary origin (e.g. [16,69,82]). Comparative mapping of specific repeats between closely related species can support or disprove the homoeology of sex chromosomes [61,69,80,103] and identify ancestral parts of multiple sex chromosomes [81]. A specific block of accumulated repeats may point to regions of suppressed recombination, breakpoints of chromosome rearrangements, or to remnants of centromeric sequences inside the fused chromosome [61,70,80]. Similarly, interstitial telomeric sequences (ITSs) i.e. telomeric repeats located by FISH inside the chromosome may signal chromosome rearrangements [104] and thus help to trace the mechanism of multiple sex chromosome formation. However, our dataset (electronic supplementary material, table S1) suggests that in eight out of 16 studied cases, ITSs have not been found in the supposed fusion points, which may reflect either their erosion or low copy number falling below the resolution of FISH. In two other species, the ITSs were found on multiple sex chromosomes but were probably not relevant to their origin [75,105]. Nevertheless, FISH with the telomeric probe is also particularly useful for determining a number of chromosomes involved in meiotic multivalents (e.g. [36,106]). More thorough characterization of repeats involved in fish multiple sex chromosome differentiation has been recently enabled by the implementation of novel bioinformatic pipelines such as RepeatExplorer as exemplified by *M. elongatus* [107].

Comparative genomic hybridization (CGH) might also assess the differentiation of multiple sex chromosomes. Simultaneous hybridization of male and female whole-genome probes to chromosome spreads may reveal differentially painted regions, which can correspond to non-recombining loci accumulating sex-limited or -enriched sequences. To date, this method has been used in diverse fishes with multiple sex chromosomes with varying success [51,52,108–110]. One drawback, however, of CGH is its low resolution as it fails to provide detailed sequence composition of the sex-specific region.

Gene content of fish multiple sex chromosomes can play an important role in their evolution. Sex chromosome–autosome fusions considerably increase the number of sex-linked genes which can contribute to adaptation and reproductive isolation (cf. [111]). Multiple sex chromosomes may thus promote the formation of pre- and post-zygotic barriers in teleosts as seen in sticklebacks [46]. Recently, it was shown that early differentiation of multiple sex chromosomes in *Drosophila miranda* was accompanied by the massive amplification of gene copies leading to their tandemly repeated arrangement on both X and Y chromosomes [112]. Similarly, the amplification of genes linked to complex multiple sex chromosome systems was observed also in wood white butterflies of the genus *Leptidea* [113], suggesting that the mechanism could be a common feature of sex chromosome differentiation. Thus, a combination of gene amplification with the frequent sex chromosome turnover observed in fishes, both possibly induced by increasing mutation load, could systematically create novel selectable variation and thus considerably increase the adaptive potential in fishes (cf. [114]).

### (b) Differentiation of teleost multiple sex chromosomes

There are major differences in the way distinct types of teleost multiple sex chromosomes degenerate. Our dataset (electronic supplementary material, table S1) shows a high prevalence of male heterogamety (*n* = 71) with no substantial degeneration of neo-Y chromosomes detectable by cytogenetic methods. In one specific case, the high heterochromatin content was already present on the ancestral Y chromosome prior to multiple sex chromosome origination [79]. However, while acknowledging the low number of known cases (*n* = 4), W chromosomes in female-heterogametic multiple sex chromosome systems accumulated heterochromatin and/or specific repeats in all cases except for the bushymouth catfish *Ancistrus dolichopterus* [16,28,60,61,64].

The reason for such disparity in the incidence and trajectory of multiple sex chromosome differentiation between male and female heterogamety is not clear. One possibility might be that fish W sex chromosomes degenerate faster in standard systems, restricting the opportunity for sex chromosome turnover. This is corroborated by hybridization patterns of W-specific chromosome probes which usually paint only a small portion of Z owing to rapid W differentiation (e.g. [18,20,62,115]), while probes from male-heterogametic systems usually paint all sex chromosomes equally in the complement (e.g. [52,109,110]).

It must be noted that the majority of cytogenetic reports documenting highly degenerated ZW sex chromosome systems are confined to the neotropical representatives of the order Characiformes, where several monophyletic and evolutionarily old systems have been described ([15–20]; electronic supplementary material, table S4), allowing the possibility of sampling bias in our general assumption. Moreover, Pennell *et al*. [116] concluded based on formal analysis conducted on data from the Tree of Sex database [13] that there are no differences in the rates of transition from homomorphic to heteromorphic sex chromosomes between XY and ZW systems.

We compiled our own datasets (electronic supplementary material, table S4; for summaries see the electronic supplementary material, Appendix S1—Appendix tables 1 and 2) using various filtering criteria (for details see the electronic supplementary material, Appendix S1) and used them to test the following hypotheses. First, we tested whether XY and ZW systems differ in sex chromosome differentiation. Fisher's exact test identified significant (*p* = 3.7 × 10^−7^) excess of homomorphic sex chromosomes in lineages with male heterogamety (65.8%) compared to lineages with female heterogamety (20.9%). We further sought to test our hypothesis that ZW systems differentiate faster in evolutionarily young sex chromosome systems. Fisher's exact test revealed that the proportion of homomorphic cases was significantly higher (*p* = 0.006) in XY systems (88.9%) than in ZW sex chromosomes (33.3%). However, the dataset for the test contained 45 male-heterogametic sex chromosome systems but only six records of female-heterogametic systems. We noted that compared to ZW systems, there are more homomorphic XY systems that escaped cytogenetic detection but were captured by other genetic or genomic approaches (electronic supplementary material, Appendix S1). We decided to compile yet another, hopefully larger, dataset using this as a proxy for level of sex chromosome differentiation. The resulting dataset contained 53 records of XY sex chromosomes with 42 homomorphic systems and nine cases of ZW sex chromosomes with four systems being homomorphic. For this dataset, Fisher's exact test also revealed a significant difference in the proportion of homomorphic sex chromosomes between male and female heterogamety (*p* = 0.0417). It should be noted that the latter dataset could be biased by genomic studies focused mainly on taxa of economic importance. Taken together, we hypothesize that rates of sex chromosome differentiation vary between male and female heterogamety and our results warrant further study.

On the mechanistic basis, one probable explanation for possible faster W sex chromosome differentiation may be heterochiasmy, i.e. the difference in rates of recombination and chiasma localization between males and females, which is indeed widespread [95,117–121]. Typically, recombination rates are higher in subterminal chromosome regions in males, while in females recombination is higher in interstitial regions, i.e. across much of the chromosome length [117]. Inversions on sex chromosomes should fix more frequently in ZW taxa under the typical recombination landscapes as selection favours rearrangements proportionally to how much they reduce recombination between sexually antagonistic and sex-determining loci [117]. Another explanation could stem from dosage-dependent male sex determination mechanisms confirmed in some female-heterogametic taxa such as the chicken [122] and the half-smooth tongue sole, *Cynoglossus semilaevis* [123,124]. In these taxa, male sex is determined by the dosage of Z-linked MSD genes. Since there is no MSD gene on a W chromosome, it is reasonable to assume that the W chromosome is functionally less constrained and under weaker purifying selection, therefore allowing faster differentiation. Our hypothesis does not exclude the possibility that the W chromosome may acquire an MSD gene secondarily, e.g. by transposition. Bearing in mind the difficulties in assembling highly degenerate sex chromosomes [125], transcriptomic studies might be more helpful in testing our hypothesis.

### (c) Possible drivers of the evolution of fish multiple sex chromosomes

The emergence of multiple sex chromosomes represents an interesting diversion from the classical scheme of sex chromosome differentiation as it may substantially change the composition, epigenetic landscape and evolutionary dynamics of these elements. Drivers of sex chromosome turnover, including the formation of multiple sex chromosomes in vertebrates, have recently been comprehensively reviewed [21,29,41,126–128]. Several different scenarios can be applied to teleost species with multiple sex chromosomes.

Our dataset (electronic supplementary material, table S1) suggests that sex chromosome–autosome fusions are by far the most prevalent mechanism giving rise to multiple sex chromosomes in teleosts. Also, the incidence of sex chromosome–autosome fusions is much higher in male-heterogametic than female-heterogametic taxa (Fisher's exact test for all data *p* = 4.4 × 10^−10^; non-parametrical ANCOVA adjusting for the total number of sex chromosomes cases *F*_1,147_ = 10.33; *p* = 0.0016; for details see the electronic supplementary material, Appendix S1 and tables S5–S6), thus following empirical evidence from other vertebrates [41,128].

Our compiled data further indicates that Y–A fusions predominate over other types of sex chromosome–autosome fusions (electronic supplementary material, table S7), as previously reported in other cold-blooded vertebrates [41]. The most plausible explanation for the observed pattern would be that sex chromosome–autosome fusions are deleterious and fixed by genetic drift [129]. According to the drift hypothesis, sex chromosome–autosome fusions should be fixed most often in sex chromosomes with the smallest effective population size, i.e. the Y and W chromosomes. Yet, the dearth of W–A fusions in vertebrates is not consistent with the drift ([41]; but cf. [130]). Furthermore, it is important to note that multiple sex chromosomes are not limited only to fishes with small or fragmented populations and low vagility but can be also found in marine lineages with high dispersal potential (e.g. [131]). Pennell *et al*. [41] concluded from their analysis that the prevalence of Y–A fusions in fishes and reptiles can be best explained by a combination of underdominance of the fusions, male-biased mutation rates for fusions and female-biased reproductive sex ratio.

Pokorná *et al*. [128] proposed that female meiotic drive could constrain the incidence of multiple sex chromosomes in female-heterogametic systems. Meiotic drive is the non-random segregation of chromosomes owing to the asymmetry of female meiosis, which results in only one functional gamete, oocyte and three polar bodies [132]. This mechanism affects all chromosomes in the complement and may also contribute to preferential fixation of certain multiple sex chromosome systems. In mammals, for instance, the XY_1_Y_2_ sex chromosome system created by X–A fusion was shown to be more prevalent in complements dominated by bi-armed chromosomes as the drive favours the bi-armed chromosome morphology. On the other hand, complements dominated by uni-armed elements displayed a higher incidence of the X_1_X_2_Y sex chromosome system originated from Y–A fusion since the Y chromosome never enters female meiosis and therefore is not affected by the drive [133]. When applied to ZW-derived multiple sex chromosomes, meiotic drive would cause a substantial sex ratio distortion in females and is therefore selected against. In our dataset with teleost species, abundance of multiple sex chromosome systems originated by rearrangements of Y chromosomes (50 out of 54 of independent origins; electronic supplementary material, Appendix S1–Appendix figure 1 and table S7), which is never involved in female meiosis, and scarcity of X–A (*n* = 2) and Z–A (*n* = 1) fusions makes any formal analysis of the effect of meiotic drive on fish multiple sex chromosome formation impossible. We therefore performed a Wilcoxon rank-sum test to assess the association of the percentage of uni-armed/bi-armed chromosomes on the emergence of sex chromosome–autosome fusions and fissions (electronic supplementary material, Appendix S1, Appendix figure 1 and table S7). The association between the type of rearrangement and the karyotype structure was not significant for either males (complete dataset; *Z* = 174, *p* = 0.128) or females (complete dataset; *Z* = 178.5, *p* = 0.096). We can conclude that the correlation between a high percentage of uni-armed chromosomes in the karyotype and fusion leading to multiple sex chromosomes is not significant in the analysed teleosts, and inversely, the proportion of bi-armed chromosomes does not correlate with fissions.

It was noted that certain chromosomes are ‘better at sex’ than others [134]. To assess whether sex chromosomes arose independently or by co-option of the same synteny block, FISH with bacterial artificial chromosomes [46,101,135], and whole-chromosome painting with probes derived from specific sex chromosomes [16,62,110,136,137] can be used. For instance, a common origin of multiple sex chromosomes was revealed in two *Oplegnathus* knifejaw species [110] and partial homoeology between different multiple sex chromosome systems was shown in sticklebacks [46,47] and tentatively also in the glass knifefishes of the genus *Eigenmannia* [138].

It has been hypothesized that fusions between sex chromosomes and an autosome enriched in sexually antagonistic genes are favoured by selection [44,139]. Simulations further suggested that sexually antagonistic selection may contribute to the elevated fixation of Y–A fusions when it evolves asymmetrically, i.e. alleles advantageous for males and detrimental for females will be maintained in the population with higher frequency than those with the opposite effect [140]. However, the results gathered so far, especially in ranid frogs, provided no support for the theoretical role of sexually antagonistic genes in the evolutionary dynamics of sex chromosomes [141,142]. Alternatively, different genes involved in the sex-determining pathway can take over the role of the MSD gene and give rise to new sex chromosomes [47,143,144]. Indeed, a handful of genes are being repeatedly co-opted as MSD in fishes [10,123,143,144] and thus may contribute to the repeated use of the same linkage groups as sex chromosomes.

Furthermore, it has been argued that hybridization between populations with different sex chromosome systems could promote sex chromosome turnover [45,145]. This is evidenced by a recent report of experimental crosses between a strain of the platyfish *Xiphophorus maculatus* with XY and the swordtail *Xiphophorus helleri* with ZW, which led to the translocation of the sex-determining region to an autosome in hybrids [145]. It is therefore reasonable to hypothesize that interaction between populations differing in their sex chromosome systems [31,59,65,70,79,83,85–89] can give rise to novel sex chromosomes including multiple sex chromosome systems.

## 5. Conclusion

It is generally accepted that multiple sex chromosomes represent evolutionarily young sex chromosome systems, which can provide insight into sex chromosome evolution. This is seemingly less important in teleosts in which sex chromosome turnovers frequently occur. However, the multiple sex chromosomes could indicate the presence of homomorphic sex chromosomes within a lineage and, as we showed, allow for interesting inferences about the evolution of fish sex chromosomes. We compiled 440 cases of sex chromosomes in teleost fishes and collected detailed information about 75 cases of multiple sex chromosomes, which correspond to 60 independent origins of these systems. We further showed that multiple sex chromosomes are over-represented in the order Perciformes. Sex chromosome–autosome fusions were the most prevalent mechanism giving rise to multiple sex chromosomes in teleosts and their incidence was much higher in male-heterogametic than female-heterogametic taxa. We further documented a striking difference in patterns of differentiation of sex chromosomes between male and female heterogamety. We hypothesized that faster W sex chromosome differentiation may constrain sex chromosome turnover as it significantly reduces the time window in which it may take place. We also showed no significant association between the formation of multiple sex chromosomes and the percentage of uni-armed chromosomes in teleost karyotypes. We highlighted the gaps in research of multiple sex chromosomes in teleosts and emphasized the need for fine-scale analyses and broad sampling. Integration of molecular cytogenetics with genomics can fill the gaps and contribute to a more complex understanding of vertebrate sex chromosome evolution.

## Data accessibility

All data and methods are provided in the electronic supplementary material.
